# Prevalence of Mental Distress and Associated Factors among Samara University Students, Northeast Ethiopia

**DOI:** 10.1155/2020/7836296

**Published:** 2020-01-24

**Authors:** Robel Tesfaye Kelemu, Alemayehu Bayray Kahsay, Kedir Y. Ahmed

**Affiliations:** ^1^Afar Regional Health Bureau, Afar National and Regional, PO Box: 28, Semera, Ethiopia; ^2^School of Public Health, College of Health Sciences, Mekelle University, Ethiopia; ^3^Department of Public Health, College of Medicine and Health Science, Samara University, PO Box: 132, Samara, Ethiopia; ^4^Translational Health Research Institute, Western Sydney University, Campbelltown Campus, NSW, Australia

## Abstract

**Background:**

Empirical findings have indicated that higher institution students experience a higher prevalence of mental distress compared to the general population. Understanding the magnitude and associated factors of mental distress in university students would be helpful to practitioners and policymakers in Ethiopia. The aim of the present study was to examine the prevalence and associated factors of mental distress among Samara university students, Northeast Ethiopia.

**Methods:**

Institution based cross-sectional study design was conducted in Samara university from December to June 2018. A simple random sampling technique was employed to select the study participants. Self-Reporting Questionnaire-20 (SRQ-20) was used to measure the mental distress of students. Multivariable logistic regression modeling was used to examine the association between sociodemographic and psychosocial factors with the mental distress of students.

**Results:**

The proportion of students with mental distress were found to be 53.2% (95% confidence interval [CI]: 48.0%, 58.0%). Female students were more likely to be mentally distressed compared to male students (adjusted odds ratio [AOR]: 4.66; 95% CI: 2.81, 7.71). Ever khat use (AOR: 3.09; 95% CI: 1.74, 5.50) and poor sleep quality (AOR: 2.23; 95% CI: 1.12, 3.66) were significantly associated with mental distress of students.

**Conclusion:**

Our study indicates that the proportion of mental distress was found to be higher among Samara university students as compared to previously published studies in Ethiopia. Female students, ever khat users and those with poor sleep quality were associated with mental distress. There is a need for evidence-based interventional strategies such as self-help measures, sleep hygiene and peer support, as well as professional mental health services as part of student health services that would be helpful to reduce the burden of mental distress of students.

## 1. Background

Mental distress is a syndrome characterized by a clinically significant disturbance in cognition, emotion regulation, or behaviour accompanied by psychological, biological, or developmental processes dysfunction [1, 2]. Empirical findings have indicated that students experience a higher prevalence of mental disorders than the general population [3]. This is, even more, higher among students of higher institutions. There are several possible explanations for the increased mental distress of university students. First, students have to make significant adjustments to college life. Second, because of the pressure of studies, there is strain placed on interpersonal relationships. Third, housing arrangements and changes in lifestyle also contribute to the stress experienced by college/university students. Furthermore, students in college experience stress related to academic requirements, support systems, and ineffective coping mechanisms [4, 5].

Mental distress can lead to temporary effects as well as consequences that affect the individual in the long term. Common consequence of college students mental distress are a feeling of being overwhelmed [4, 5]; inability to concentrate and to focus the attention on a certain task which can result in being unable to answer questions in an exam [4, 6, 7], and finally may result in withdrawal from their college or university. In the long term, if mental distress is perceived as negative and excessive, it can result in physical and psychological impairment [8]. Studies showed that excessive stress is associated with both sleep problems and substance use, and mental health symptoms in young adolescents [6, 7].

In Ethiopia, however, despite more than one-third of the university students affected by mental distress at least once during their campus life, mental health has been one of the most disadvantaged health programs in higher institutions, both in terms of facilities and trained manpower [9–12]. To institutionalize policies and strategies for intervention and control of the mental distress of students, understanding the magnitude and predictors of mental distress in university students would be helpful to practitioners and policymakers in Ethiopia. Moreover, Samara university is located in one of the hottest areas in the country which might exacerbate the living condition of students. Therefore, the aim of this study was to examine the prevalence and associated factors of mental distress among Samara university students in Ethiopia.

## 2. Methods

### 2.1. Study Design and Study Setting

Institution based cross-sectional study design was used to assess the prevalence and associated factors of mental distress among Samara University students from February to March 2018. The study was conducted in Samara university, which is found at Semera-Logia town of Afar National and Regional State (ANRS). Semera-Logia town of Afar National and Regional State (ANRS) is located 583 km away from the capital of Ethiopia, Addis Ababa. In the 2017/2018 academic year, a total of 8,777 students were enrolled in regular, extension, the summer and post-graduate programs [13].

### 2.2. Source and Study Population

All regular Samara university students who were registered during the 2017/2018 academic year were the source population. Students selected by simple random sampling technique were the study population. Students who are unable to see and were out of the campus during the data collection period were excluded from the study.

### 2.3. Sample Size Determination and Sampling Procedure

A single population proportion formula assuming 95% confidence interval (CI), 5% margin of error, and 49.1% proportion of mental distress [14] was used to calculate the sample size. Considering a non-response rate of 10%, the final sample size of the study was found to be 422. After we select one department from each college, we used simple random sampling technique (table of random number) to select students from each department.

### 2.4. Data Collection

The data were collected using a self-administered questionnaire with four parts. First, a socio-demographic characteristic of students was asked. Second, the Self-Reporting Questionnaire (SRQ_20_) was used to measure the prevalence of mental distress. SRQ_20_ is originally developed by the World Health Organization (WHO) [15] designed to indicate common mental disorders or mental distress. The tool was validated in low and middle-income countries (including Ethiopia) [15]. In this study, students who are found to have eight or more symptoms of SRQ_20_ questions in the last four weeks were considered as having mental distress. This cutoff point was used based on a validation study of the questionnaire which gave the highest sensitivity and specificity [9]. Third, ever and current substance use (i.e. Alcohol, khat, and cigarette) were asked. The last part of the questionnaire measured sleep quality and patterns of students using the Pittsburgh Sleep Quality Index. The PSQI is an effective instrument used to measure the quality and patterns of sleep in adults. It differentiates “poor” from “good” sleep by measuring seven areas (components) : subjective sleep quality, sleep latency, sleep duration, habitual sleep efficiency, sleep disturbances, use of sleeping medications, and daytime dysfunction over the last month [10].

One Psychologist as a supervisor and three diploma nurses as data collector participated in the data collection process. The distribution of the questionnaire was conducted to students while they were in the classroom.

### 2.5. Data Quality Control

Training was provided for the data collection team on the objective and overall data collection procedures on a day before the pretest. The pretest was also conducted on 10% of students of Samara university from non-selected departments. The daily meeting was conducted among the principal investigator, supervisor and data collectors to check completeness and clarity of the questionnaire and to resolve unanticipated problems.

### 2.6. Operational Definitions

*Mental distress:* Having eight or more symptoms of the 20 SRQ_20 _questions in the last four weeks. *Current substance use:* a history of substance use (for non-medical purposes) in the last four weeks. *Ever substance use:* using the specified substance (for non-medical purposes) even once in their lifetime. Students who scored >5 for PSQI were categorized under *poor sleep quality* otherwise they were catagorized under *good sleep quality* [10].

### 2.7. Data Processing and Analysis

After checking the consistency and completeness of the questionnaires, the data were entered into Epi info version 7.1.1.14, and then exported to SPSS version 20 for further analysis. Frequency, mean, and standard deviation were employed for descriptive analysis. Multivariable logistic regression modeling was used to examine the relationship between sociodemographic and psychosocial factors with the mental distress of students. Adjusted Odds Ratio with a 95% confidence interval was used as a measure of association. A *P*-value of <0.05 was used to declare statistical significance.

### 2.8. Ethical Consideration

Ethical clearance was obtained from the Samara university College of Medical and Health Sciences Ethical Review Committee. Voluntary informed consent was also obtained from each study participant after the purpose and importance of the study was communicated. The name of the participant and any personal identifier were omitted from the questionnaire to ensure confidentiality.

## 3. Results

### 3.1. Socio-Demographic and Academic Characteristics

Out of 404 respondents, 230 (56.9%) of them were male, and 255 (63.1%) of them were less than or equal to 21 years of age. The majority of the students were single in their marital status [380 (94.1%)], and 219 (54.2%) of them were originated from urban areas. A family history of mental illness was reported in 99 (24.5%) of students. About 97 (24.0%) of them were from the College of Engineering and Technology and 152 (37.6%) of them were second-year students (Table 1).

### 3.2. Psycho-Social Characteristics

The current use of khat, alcohol, and tobacco was reported in 64 (15.4%), 86 (21.3%) and 16 (4%) of students (Table 2). The majority (77.2%) of them reported the sleeping duration of fewer than six hours. One hundred sixty-nine (41.8%) of students reported sleep latency and day time dysfunction of less than once in a week. Sleep medication was reported by 45 (11.1%) of students. Overall, 319 (79%) of students were classified as having poor sleep quality (total PSQI score >5) (Table 2).

### 3.3. Prevalence of Mental Distress

Prevalence of mental distress among students was found to be 53.2% [95% confidence interval [CI]: 0.48, 0.58]. About 58.1% and 41.7% of female and male students had mental distress respectively.

### 3.4. Factors Associated with Mental Distress

In multivariable logistic regression, female sex, ever use of khat, and sleep quality were associated with a higher odds of mental distress among students. The *P*-value of the Hosmer and Lemshow model fitness test was 0.93. The odds of mental distress of female students was significantly higher (adjusted odds ratio [AOR]: 4.67; 95% CI: 2.81, 7.71) as compared to male students. Students who reported ever use of khat had higher odds (AOR: 3.09; 95% CI: 1.74, 5.50) of mental distress compared to their counterparts. The prevalence of mental distress was significantly higher (AOR: 2.02; 95% CI: 1.12, 3.66) among students with poor sleep quality than those who had poor sleep quality (Table 3).

## 4. Discussion

The present study investigated the prevalence and associated factors of mental distress among Samara University students. Our findings showed that the prevalence of mental distress among Samara University students was found to be 53.2%, indicating the higher magnitude of mental distress among higher education students in Ethiopia. The proportion of mental distress found to be higher than previously published studies conducted among university students from Adama, Gondar, and Hawassa [12, 16, 17]. The possible explanation for the difference with the above studies can be that Samara University is located in one of the hottest areas of the country which increases student's risk of heat exhaustion and heat stress [18]. Additionally, poor infrastructure and lack of recreational facilities either inside or outside of the campus could also probably explain the observed difference in the magnitude of mental distress. The proportion of mental distress was also higher than studies conducted in higher-income countries such as France (25.7%) [19], Norway (22.9%) [20], Iceland (22.5%) [21] and Australia (19.2%) [22]. The findings of this study suggested the need for interventions targeted to reduce the mental distress of Samara University students in Ethiopia.

International evidence indicates that the magnitude of mental distress is higher in female students than male students [19, 20]. Consistent with this evidence, our study showed that the odds of mental distress was found to be higher among female students compared to male students. This finding was also supported by previously published studies in Australia [22, 23], France [24], Norway [20] and Turkey [25]. The susceptibility to stressors due to domestic violence and hormonal changes during menstruation could probably explain the higher prevalence of mental distress among female students [26]. Additionally, the structural determinants of mental health such as income and social roles and rank of women may explain the observed relationship between female students and mental distress. There is a need for a public health primary prevention approach and gender-specific interventions that address gender-specific risk factors in Ethiopia.

Researchers have indicated the bi-directional relationships between substance use and common mental disorders of students [27, 28]. Our study also supported this relationship which showed the positive association between ever use of khat and mental distress of students. Similarly, previously published studies in Ethiopia also reported that substance use was associated with increased risk to mental distress of students [12, 29]. This result may be explained by the fact that people may use psychoactive substances as a self-regulation strategy to alleviate the distressful experience [28]. Another possible explanation for the observed association between substance use and mental distress could be that people with substance use and mental disorder may have an overlapping genetic susceptibility to both disorders [30]. The evidence from this study suggests that it is imperative that practitioners and policymakers work collaboratively to establish multi-pronged strategies to reduce the co-occurring substance use and mental distress of students.

The present study demonstrated that students who had poor sleep quality were more likely to experience mental distress as compared to those who had good sleep quality. This finding was supported by similar studies conducted among university students [31–33]. The relationship between poor sleep quality and mental distress can be explained by that students with sleep disturbances are more likely to complain a high level of stress, which might, in turn, be changed to mental distress [34]. There are, however, other possible explanations. The sleeping disturbance can be either a cause or a symptom of mental distress or simply co-morbidity [32]. We recommend high-quality longitudinal studies that might be helpful to investigate the relationship between mental distress and sleep quality of university students.

This study has some important limitations that should be kept in mind when interpreting the results. First, the cross-sectional nature of the study design may not allow confirming a definitive cause and effect relationship. Second, it is important to bear in mind that SRQ_20_ is a screening instrument in measuring the mental distress of students. Nevertheless, the findings of this study can be used as a first step to understand the current situation of mental distress among university students. Third, the scope of this study was limited in terms of measuring the distressful experience of students. We recommend for qualitative investigations that explore the experience and perception of students towards the distressful experience. Last, the study may be prone to recall bias since the data were collected based on self-reported information. Despite the above limitation, the use of a validated standardized instrument can be considered as a potential strength of this study.

## 5. Conclusion

The result of the present study shows that more than half of Samara university students were mentally distressed. This proportion was higher as compared to similar studies conducted in Adama, Gondar and Hawassa university students. Being female in sex, ever use of khat and poor sleep quality were independent predictors of student's mental distress. We recommended that students mental distress needs due attention and remedial action from both government and non-governmental organizations and any program aimed at preventing mental distress of students. For example, evidence-based interventional strategies such as self-help measures, sleep hygiene, and peer support, as well as professional mental health services as part of student health services, would be helpful to reduce the burden of mental distress of students.

## Figures and Tables

**Table 1:** Socio-demographic and academic characteristics of Samara university students, Northeast Ethiopia, 2018 (*n* = 404).

Variables	Frequency	Percentage (%)
*Sex*		
Male	230	56.9
Female	174	43.1

*Age*		
≤21 years	255	63.1
>21 years	149	36.9

*Marital status*		
Single	380	94.1
Married	18	4.5
Divorced	6	1.5

*Religion*		
Orthodox	242	59.9
Muslim	102	25.2
Protestant	51	12.6
Other	9	2.2

*Origin of residence*		
Rural	219	54.2
Urban	185	45.8

*College*		
Engineering and technology	97	24.0
Veterinary medicine	55	13.6
Business and economics	51	12.6
Law school	50	12.4
Medical and health sciences	49	12.1
Natural and computational science	41	10.1
Social science and humanity	35	8.7
Dryland agriculture	26	6.4

*Year of study*		
1^st^ year	98	24.3
2^nd^ year	152	37.6
3^rd^ year	83	20.5
4^th^ year	15	3.7
5^th^ year	46	11.4
6^th^ year	10	2.5

*CGPA*		
>3.5	76	18.8
3.00–3.49	129	31.9
2.50–2.99	121	30.0
2.00–2.49	77	19.1
<1.99	1	0.2

*Family history of mental distress*		
Yes	99	24.5
No	305	75.5

CGPA: Cumulative grade point average.

**Table 2:** Distribution of psychosocial factors in Samara university students, Northeast Ethiopia, 2018 (*n* = 404).

Variables	Frequency (*n* = 404)	Percentage (%)
*Sleep duration [mean (+SD) = 5.3(±0.96)]*		
Greater than 7 hours	50	12.4
6-7 hours	42	10.4
5-6 hours	181	44.8
<5 hours	131	32.4

*Sleep latency*		
Not during the past month	51	12.6
Less than once a week	169	41.8
Once or twice a week	143	35.4
Three or more times a week	41	10.1

*Day time dysfunction*		
Not during the past month	118	29.2
Less than once a week	169	41.8
Once or twice a week	91	22.5
Three or more times a week	26	6.4

*Sleep efficiency*		
>85%	151	37.4
75–84%	87	21.5
65–74%	92	22.8
<65%	74	18.3

*Subjective sleep quality*		
Very good	89	22
Fairly good	122	30.2
Fairly bad	144	35.6
Very bad	48	11.9

*Sleep disturbance*		
Not during the past month	119	29.5
Less than once a week	107	26.5
Once or twice a week	100	24.8
Three or more times a week	78	19.3

*Use of sleep medication*		
Not during the past month	359	88.9
Less than once a week	25	6.2
Once or twice a week	17	4.2
Three or more times a week	3	0.7

*Sleep quality [mean (SD) = 7.91(±3.57)]*		
Good sleep quality	85	21
Poor sleep quality	319	79

*Ever use of khat [*n* = 404]*		
Yes	112	27.7
No	292	72.3

*Current use of khat [*n* = 112]*		
Yes	64	57.1
No	48	42.8

*Frequency of khat chewing [*n* = 64]*		
Once in a week	18	28.1
Two-three times in a week	31	48.4
More than three times a week	15	23.4

Ever drink alcohol		
Yes	154	38.1
No	250	61.9

Currently drinking alcohol		
Yes	86	55.8
No	68	44.2

Frequency of alcohol drinking		
Once in a week	46	53.5
Two-three times in a week	32	37.2
More than three times a week	8	9.3

Ever smoke tobacco products		
Yes	37	9.2
No	367	90.8

Current smoker		
Yes	16	43.2
No	21	56.7

**Table 3:** Factors associated with mental distress of Samara university Students, Northeast Ethiopia, 2018 (*n* = 404).

Variable	Mental distress		ORs with 95% CI	
	Yes	No	Crude	Adjusted

*Sex*				
Male	90	140	1.00	1.00
Female	125	49	3.97 (2.6, 6.06)	**4.66 (2.81, 7.71)**

*Year of study*				
1^st^ year	80	18	7.16 (3.91, 13.12)	4.28 (0.85, 21.466)
2^nd^ year	76	76	1.61 (1.02, 2.54)	0.78 (0.17, 3.65)
≥3^rd^ year	59	95	1.00	1.00

*Family history of mental illness*				
Yes	65	34	1.98 (1.23, 3.17)	1.69 (0.97, 2.96)
No	150	155	1.00	1.00

*Ever used khat*				
Yes	70	41	1.74 (1.11, 2.73)	**3.09 (1.74, 5.5)**
No	145	148	1.00	1.00

*Ever used alcohol*				
Yes	73	81	0.69 (0.58, 1.03)	0.9 (0.55, 1.47)
No	142	108	1.00	1.00

*Sleep quality and quantity*				
Good sleep quality	32	53	1.00	1.00
Poor sleep quality	183	136	2.23 (1.36, 3.66)	**2.02 (1.12, 3.66)**

ORs = Odds ratio. The bold values indicate statistical significance.

## Data Availability

The data set will not be shared in order to protect the participants' identities.

## Abbreviations

ANRS: Afar national and regional state
AOR: Adjusted odds ratio
CI: Confidence interval
CGPA: Cumulative grade point average
PSQI: Pittsburgh sleep quality index
SRQ: Self-reporting questionnaire
WHO: World Health Organization.
